# Dysphagia Lusoria Revealing Bicuspid Aortic Stenosis With an Aberrant Right Subclavian Artery and Contralateral Subclavian Aneurysm

**DOI:** 10.7759/cureus.112224

**Published:** 2026-07-07

**Authors:** Amin Ullah, Noman Rahim, Izaz Iqbal, Diyan Muhammad, Muhammad Asad B Awan

**Affiliations:** 1 Cardiac Surgery, National Institute of Cardiovascular Diseases (NICVD), Karachi, PAK

**Keywords:** aberrant subclavian artery, bicuspid aortic valve, congenital arch anomaly, dysphagia lusoria, hybrid surgical repair, subclavian artery aneurysm

## Abstract

Aberrant right subclavian artery is the most common congenital anomaly of the aortic arch and may present with dysphagia lusoria due to esophageal compression. Although bicuspid aortic valve disease is commonly associated with aortopathy, its coexistence with an aberrant right subclavian artery and a contralateral subclavian artery aneurysm is exceedingly rare. We report the case of a 33-year-old male presenting with exertional dyspnea, chest pain, presyncope, and intermittent dysphagia to solids. Echocardiography demonstrated severe bicuspid aortic stenosis with a mean gradient of 95 mmHg and a peak velocity of 7.6 m/s. Computed tomography angiography revealed a retroesophageal aberrant right subclavian artery and a 34 mm aneurysm of the left subclavian artery. Following multidisciplinary discussion, the patient underwent a single-stage hybrid procedure consisting of right carotid-subclavian bypass and translocation of the aberrant vessel via a supraclavicular approach, followed by mechanical aortic valve replacement through median sternotomy. The postoperative course was complicated by transient complete heart block, which resolved spontaneously before discharge. Follow-up imaging demonstrated a patent bypass graft and stable aneurysm size, while dysphagia resolved completely. This case highlights the importance of investigating dysphagia in young adults with congenital valvular disease and demonstrates the value of individualized hybrid surgical strategies in complex arch pathology. The management of a contralateral subclavian artery aneurysm in the setting of bicuspid aortic valve aortopathy remains a clinical dilemma without clear guideline thresholds.

## Introduction

Bicuspid aortic valve is the most common congenital cardiac valvular anomaly and is frequently associated with progressive valvular dysfunction and aortopathy [1,2]. Aberrant right subclavian artery, also known as arteria lusoria, represents the most common congenital anomaly of the aortic arch, occurring in approximately 0.5%-2% of the population [3]. In this anomaly, the right subclavian artery arises distal to the left subclavian artery and most commonly follows a retroesophageal course toward the right upper extremity.

Although an aberrant right subclavian artery is often asymptomatic, some patients develop dysphagia lusoria due to compression of the esophagus. Symptoms may manifest later in adulthood because of progressive vascular rigidity, aneurysmal degeneration, or age-related mediastinal changes [4]. Dysphagia is the most common presenting symptom in adults with symptomatic arteria lusoria and may be accompanied by chest discomfort, cough, or weight loss [5]. The coexistence of severe bicuspid aortic stenosis with symptomatic aberrant right subclavian artery and contralateral subclavian artery aneurysm is exceedingly uncommon and presents significant diagnostic and operative challenges.

The association between bicuspid aortic valve disease and aortic arch anomalies may reflect shared embryologic mechanisms involving abnormal neural crest cell migration and aberrant vascular remodeling during development of the outflow tract and aortic arch system [6,7]. However, the simultaneous presence of critical bicuspid aortic stenosis, dysphagia lusoria, and contralateral subclavian artery aneurysm has rarely been described. We report a young adult successfully managed using a single-stage hybrid surgical approach.

## Case presentation

A 33-year-old male with no prior history of cardiovascular disease presented with progressive exertional dyspnea, chest pain, intermittent presyncope, and dysphagia to solids. Symptoms had progressively worsened over several months prior to presentation. Dyspnea was classified as New York Heart Association class III, while chest pain was categorized as Canadian Cardiovascular Society class III angina.

Physical examination demonstrated a harsh ejection systolic murmur radiating to the carotids. Peripheral pulses were symmetrical, and there were no signs of heart failure. Laboratory investigations were unremarkable. Transthoracic echocardiography demonstrated preserved left ventricular systolic function with an ejection fraction of 55%. A bicuspid aortic valve with severe calcific stenosis was identified. Hemodynamic measurements revealed a mean transvalvular gradient of 95 mmHg and peak velocity of 7.6 m/s, consistent with critical aortic stenosis.

**Figure 1:**
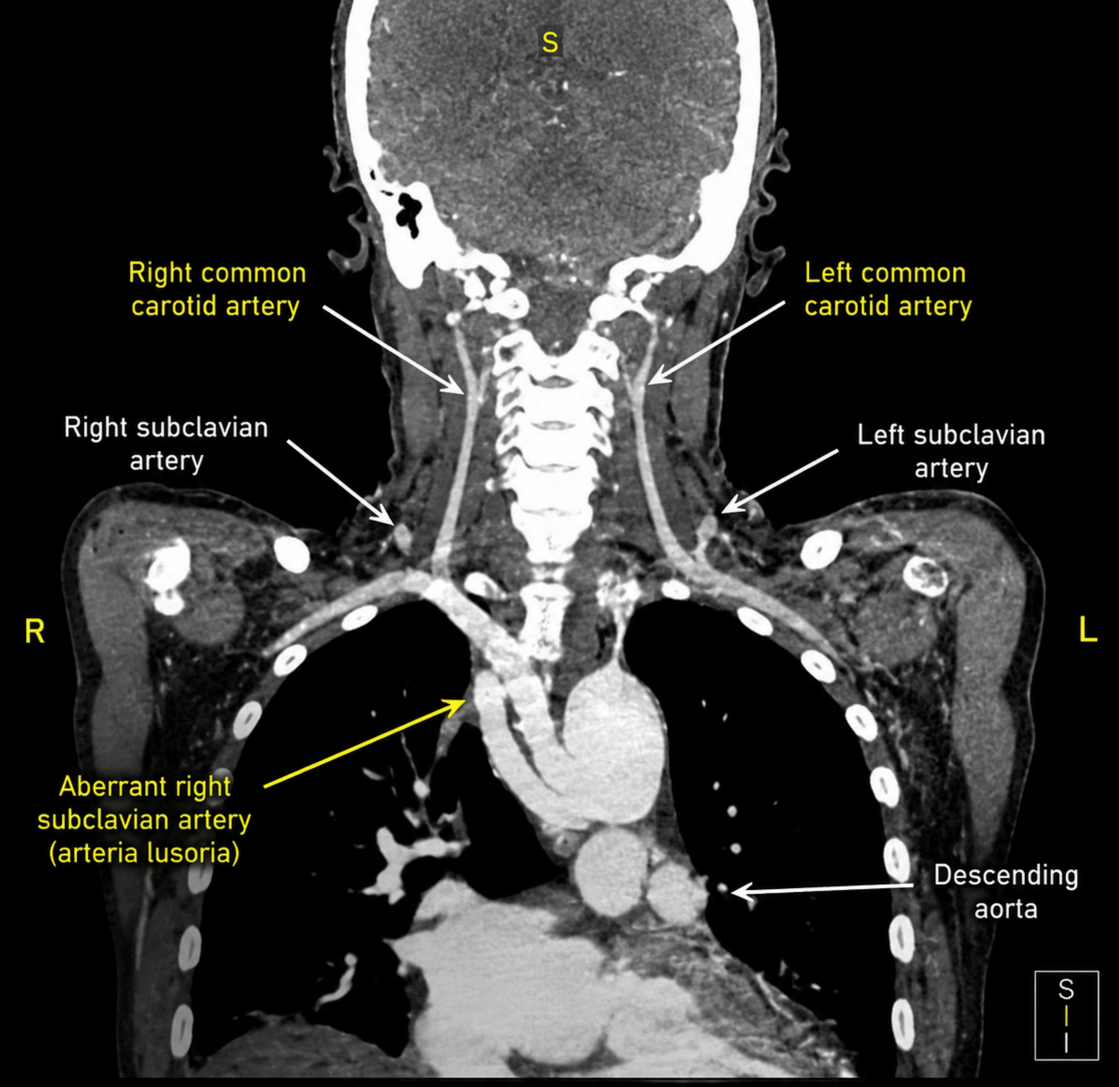
Coronal computed tomography angiography demonstrating an aberrant right subclavian artery (arteria lusoria) arising distal to the left subclavian artery.

**Figure 2:**
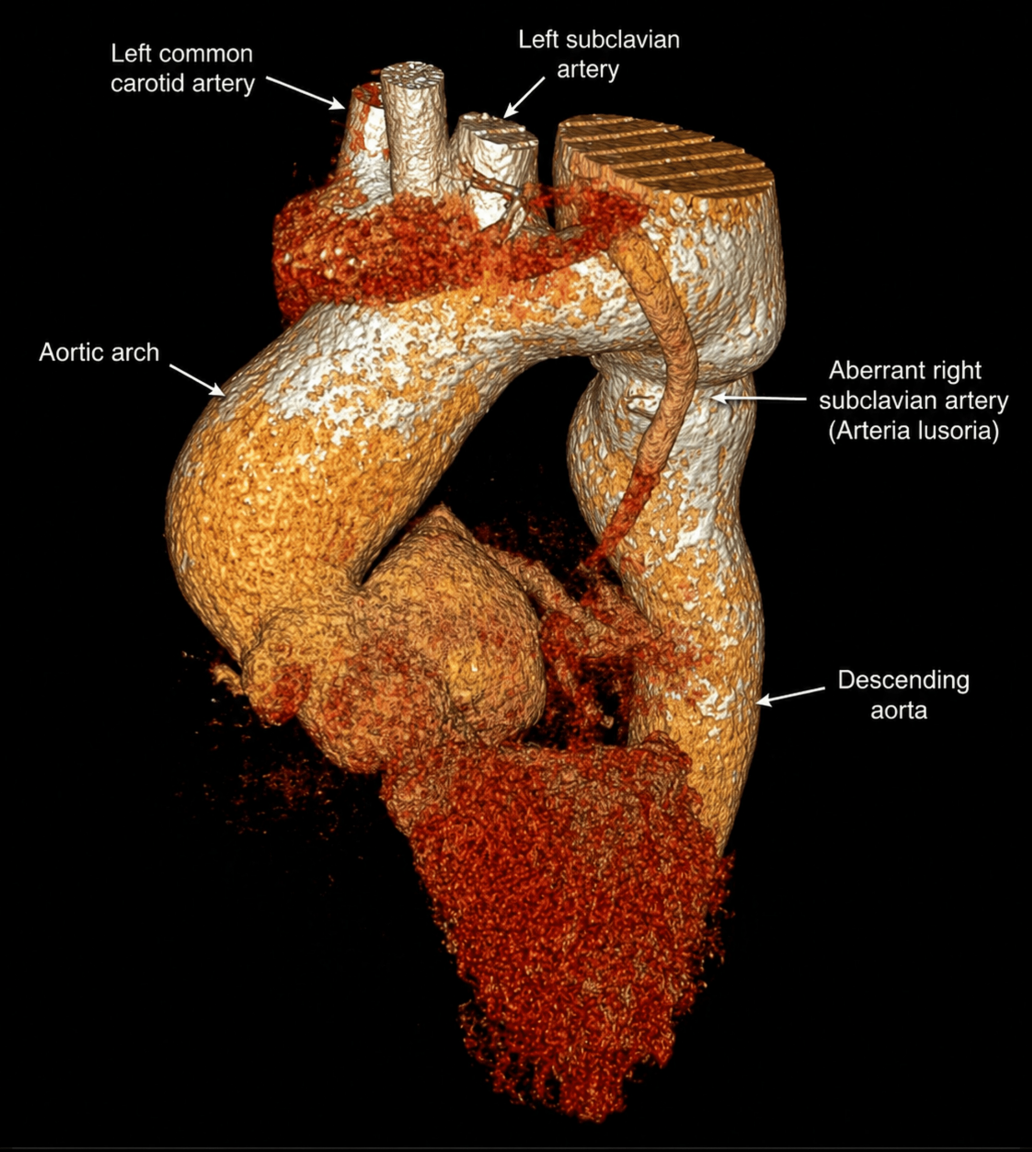
Three-dimensional computed tomography angiography reconstruction demonstrating retroesophageal aberrant right subclavian artery (arteria lusoria) with associated contralateral subclavian artery aneurysm.

**Figure 3:**
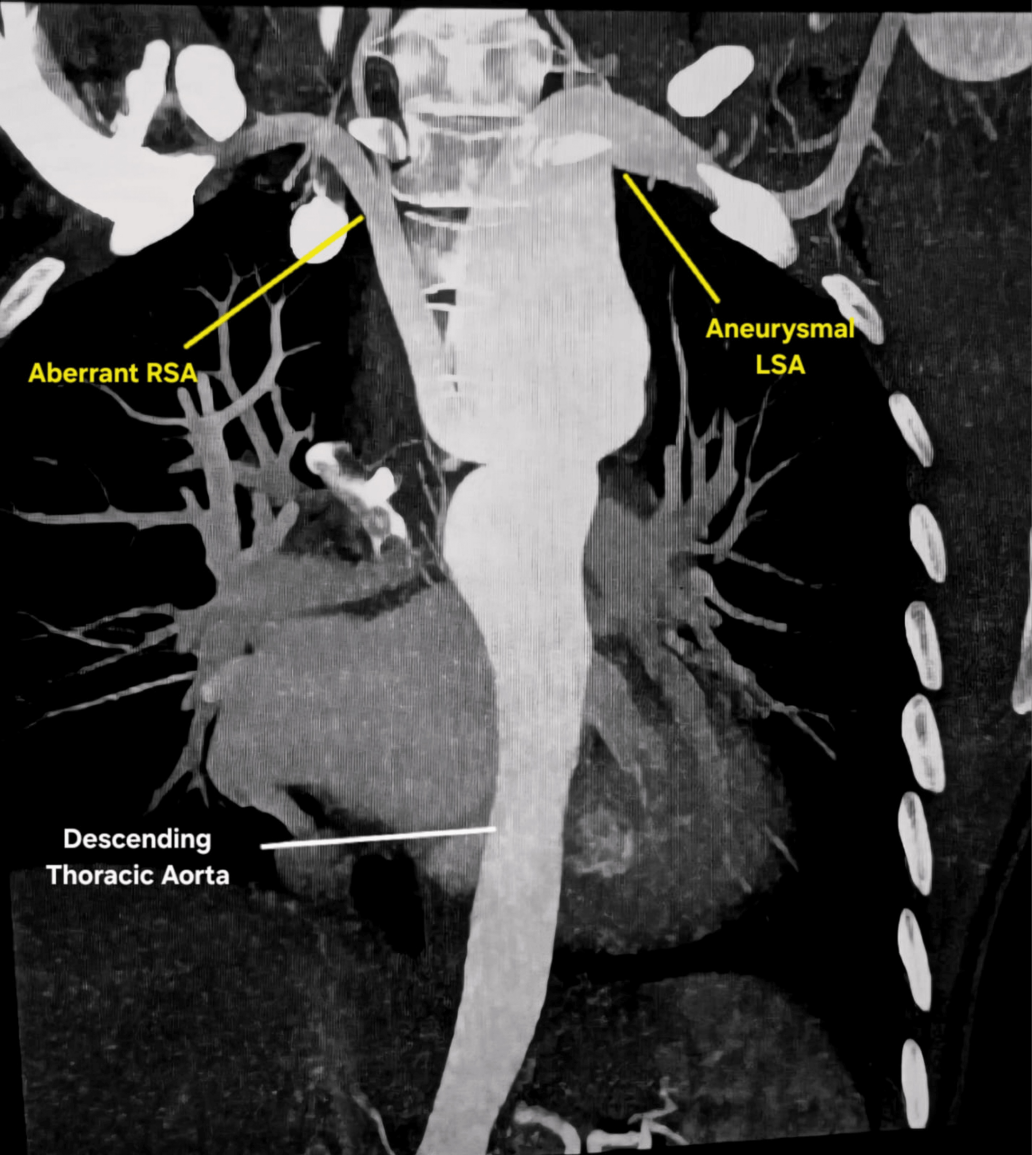
Coronal CT angiography showing aneurysmal dilatation (~34 mm) of the left subclavian artery (LSA) arising from the aortic arch. There is also an anomalous right subclavian artery (RSA) (arteria lusoria).

Computed tomography angiography of the thorax revealed a left-sided aortic arch with an aberrant right subclavian artery arising distal to the left subclavian artery. The vessel followed a retroesophageal course toward the right upper extremity, explaining the patient's dysphagia symptoms. Additionally, aneurysmal dilatation of the left subclavian artery measuring approximately 34 mm was identified (Figures 1-3). No significant ascending aortic dilatation was noted.

The patient's case was discussed in a multidisciplinary heart valve and aortic team meeting. Given the patient's young age, severe symptomatic aortic stenosis, symptomatic aberrant right subclavian artery, and associated aneurysmal pathology, a single-stage hybrid strategy was selected.

Surgical technique

The procedure was performed under general anesthesia in two steps.

Step 1: Right Supraclavicular Approach for Aberrant Right Subclavian Artery Reimplantation

**Figure 4:**
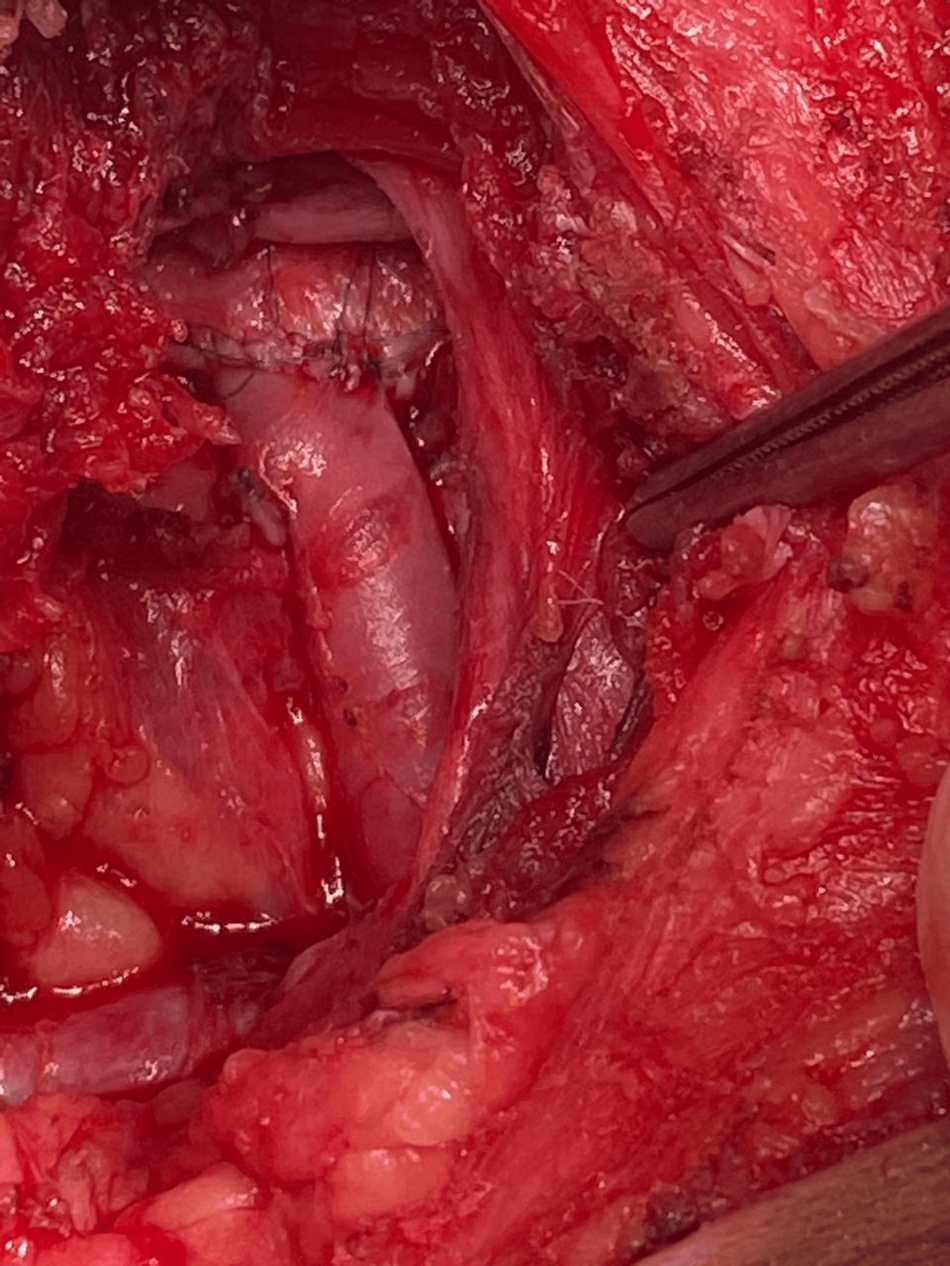
Intraoperative image demonstrating end-to-side anastomosis of the aberrant right subclavian artery to the right common carotid artery through a right supraclavicular approach.

A right supraclavicular incision was made to expose the right common carotid artery and aberrant right subclavian artery. The aberrant right subclavian artery was carefully mobilized to the level of the vertebral artery. After systemic heparinization, an end-to-side anastomosis of the aberrant vessel to the right common carotid artery was performed, and the distal stump was closed (Figure 4).

The supraclavicular approach was intentionally selected despite planned median sternotomy because it provided superior exposure and vascular control of the supra-aortic vessels while avoiding extensive deep mediastinal dissection. Exposure of the distal subclavian artery through sternotomy alone would have been technically challenging and associated with increased risk of injury to surrounding mediastinal structures.

Step 2: Median Sternotomy for Aortic Valve Replacement

**Figure 5:**
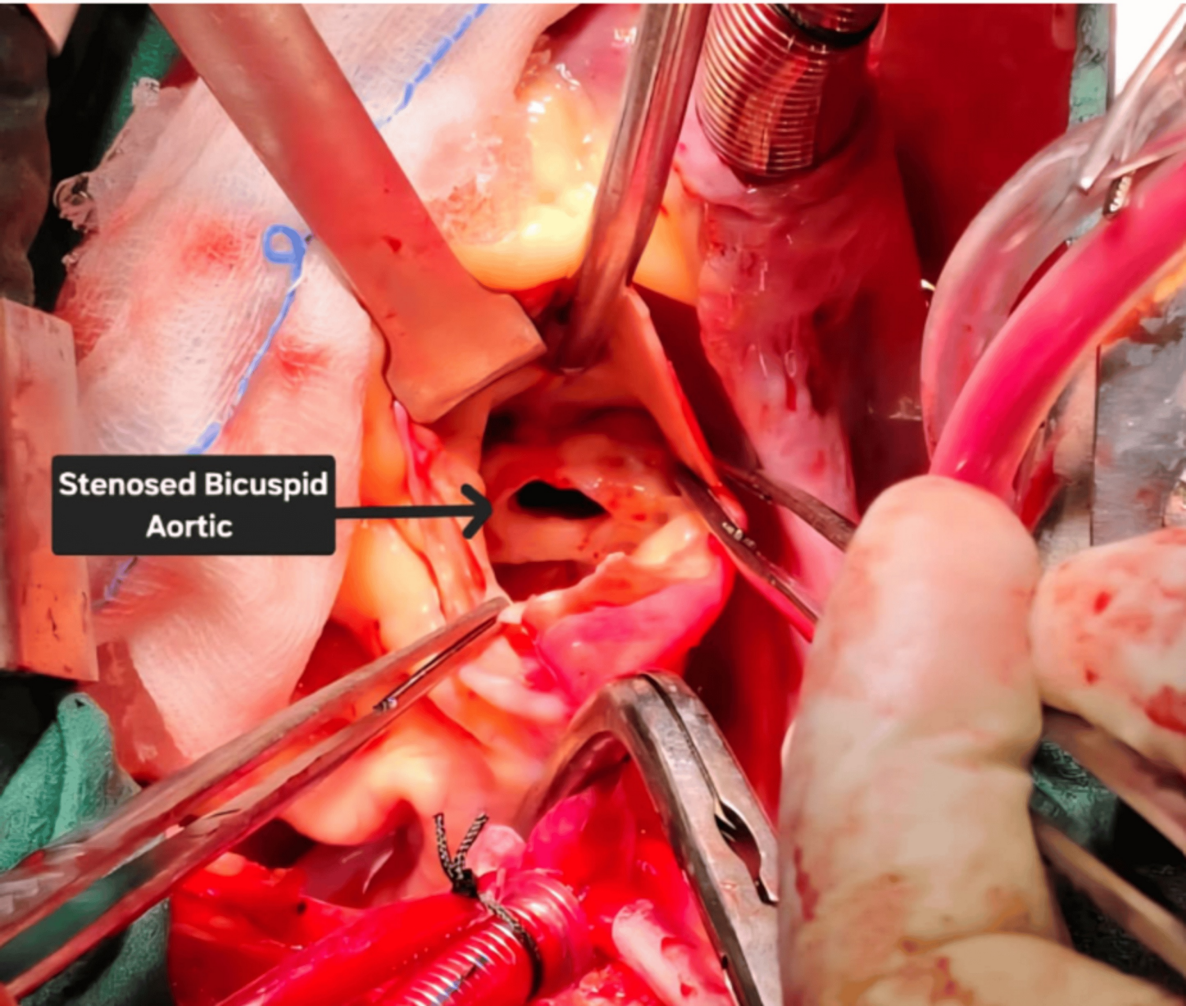
Intraoperative image demonstrating a severely stenotic bicuspid aortic valve with characteristic fish-mouth appearance during surgical aortic valve replacement.

Following completion of the supra-aortic reconstruction, median sternotomy was performed. Standard cardiopulmonary bypass was established. The aorta was cross-clamped, a left ventricular vent was placed, and ostial cardioplegia was administered. Decalcification and valvectomy were performed. A 25 mm St. Jude Medical mechanical valve (Abbott, St. Paul, MN, USA) was implanted using interrupted, pledgeted, non-everting sutures. The aortotomy was closed, and standard de-airing maneuvers were performed (Figure 5).

Intraoperatively, the aberrant right subclavian artery was confirmed to arise from the distal aortic arch with a retroesophageal course. The left subclavian artery aneurysm was noted but not addressed during this procedure, as it was asymptomatic and below the conventional threshold for intervention.

**Figure 6:**
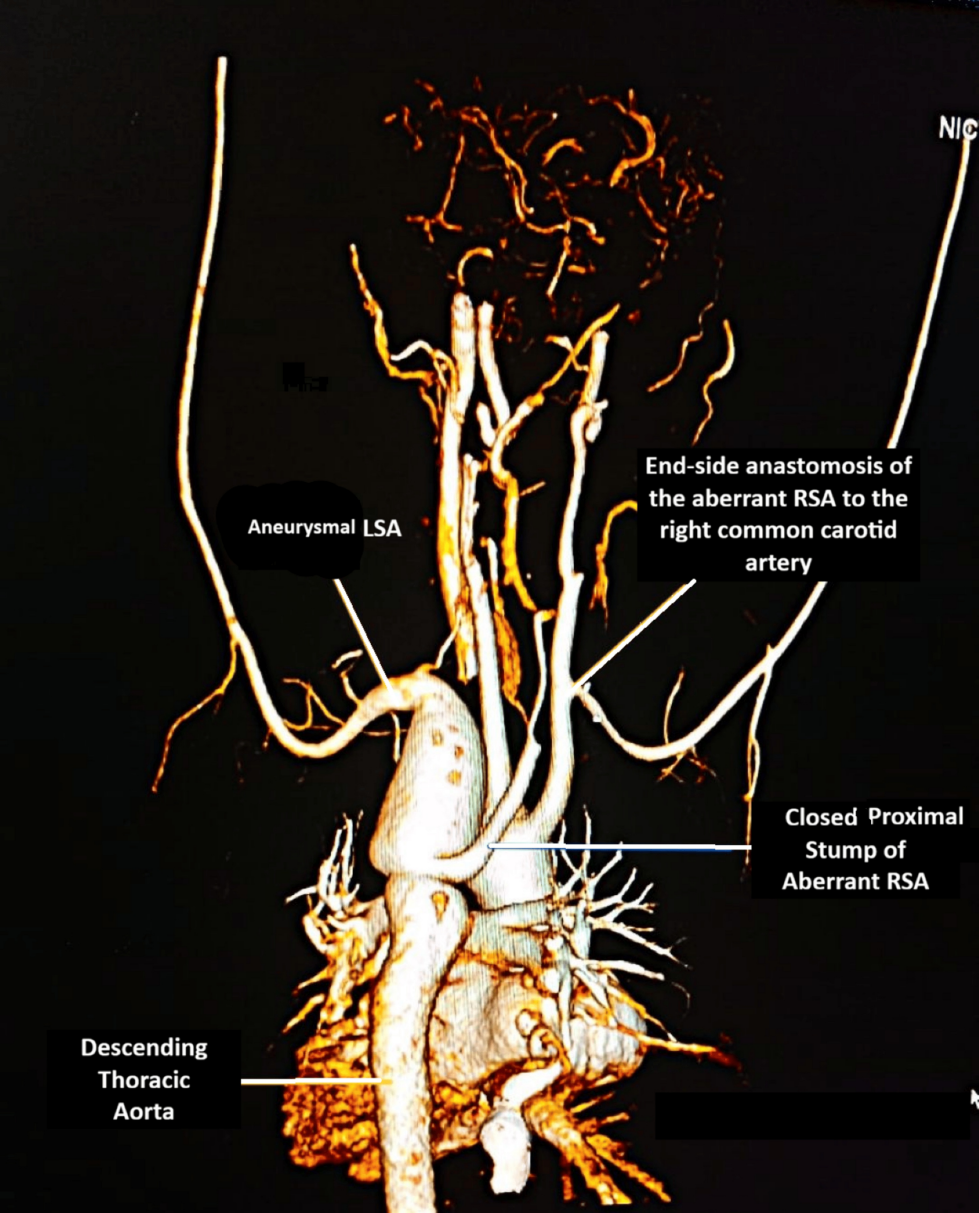
Postoperative three-dimensional computed tomography angiography reconstruction demonstrating successful end-to-side translocation of the aberrant right subclavian artery (RSA) to the right common carotid artery with ligation of the proximal aberrant stump. LSA: left subclavian artery

Post-bypass transesophageal echocardiography demonstrated a normally functioning prosthetic valve with no paravalvular leak and good biventricular function (Figure 6).

Postoperative course and outcome

On the first postoperative day, the patient developed atrioventricular dissociation progressing to complete heart block. Temporary epicardial pacing leads were placed. The conduction disturbance was transient and resolved spontaneously before discharge, and no permanent pacemaker was required. The patient was discharged in normal sinus rhythm with a therapeutic international normalized ratio (INR).

At two-month follow-up, the patient was clinically stable with occasional exertional dyspnea. Postoperative computed tomography angiography demonstrated a patent right carotid-subclavian bypass with good flow. The left subclavian artery aneurysm remained stable at 34 mm. The patient's dysphagia resolved completely following surgery.

Management of the residual left subclavian artery aneurysm

Current guidelines for aortic arch anomalies suggest that intervention for Kommerell diverticulum may be reasonable when the diverticulum orifice exceeds 3 centimeters, or the combined diameter of the diverticulum and adjacent aorta exceeds 5 centimeters. However, no specific guidelines exist for isolated subclavian artery aneurysms in patients with bicuspid aortic valve aortopathy. After shared decision-making with the patient, a conservative approach with serial imaging surveillance was adopted. The patient will undergo annual computed tomography angiography to monitor for aneurysm enlargement.

## Discussion

Clinical significance

Dysphagia lusoria is frequently underdiagnosed. The diagnosis is often delayed because symptoms are nonspecific and may mimic more common esophageal disorders, emphasizing the importance of maintaining a high index of suspicion in patients with unexplained dysphagia [8]. Approximately 10% of patients with an aberrant right subclavian artery experience dysphagia, typically worse with solids [3]. In our patient, dysphagia was the key clinical clue that prompted further imaging and led to the diagnosis. This case reinforces the importance of considering aortic arch anomalies in young patients with valvular disease presenting with atypical compressive symptoms. Computed tomography angiography with three-dimensional reconstruction remains the imaging modality of choice for defining arch anatomy, identifying associated aneurysmal disease, and facilitating operative planning [9].

Rarity of the association

The simultaneous presence of bicuspid aortic valve, critical aortic stenosis, a symptomatic aberrant right subclavian artery with dysphagia lusoria, and a contralateral subclavian artery aneurysm is exceptionally rare. While bicuspid aortic valve affects 1% to 2% of the population and aberrant right subclavian artery occurs in 0.5% to 1%, their coexistence has been reported only in isolated cases [4]. The addition of a contralateral subclavian aneurysm makes this presentation particularly unusual. To our knowledge, this specific combination has not been previously reported.

Surgical implications

Management of symptomatic aberrant right subclavian artery has evolved from open thoracotomy to hybrid techniques combining cervical revascularization with endovascular or open exclusion [6,7,10]. A systematic review demonstrated favorable outcomes across all approaches [7]. In our patient, a single-stage hybrid strategy combining right carotid-subclavian bypass via a supraclavicular approach with median sternotomy for aortic valve replacement allowed simultaneous treatment of both vascular and valvular pathology under a single anesthetic.

Management dilemma

The residual 34 mm left subclavian artery aneurysm presents a therapeutic challenge. Current guidelines provide limited recommendations for isolated subclavian artery aneurysms, particularly in bicuspid aortic valve aortopathy [11,12]. Published experience with Kommerell diverticulum and aberrant subclavian aneurysms suggests that intervention thresholds remain heterogeneous, with treatment decisions frequently individualized according to aneurysm size, symptoms, and associated arch pathology [13]. Bicuspid aortic valve is associated with intrinsic wall abnormalities that may predispose to more aggressive aneurysm behavior [4]. After multidisciplinary discussion, we elected conservative management with annual computed tomography angiography surveillance. The patient will be monitored for aneurysm enlargement, thrombosis, or rupture.

Embryological link

Both bicuspid aortic valve and aortic arch anomalies share a common developmental origin. Cardiac neural crest cells contribute to both outflow tract septation and aortic arch remodeling [14,15]. The coexistence of these abnormalities supports a shared neural crest-mediated embryologic defect.

Postoperative conduction abnormalities

The transient complete heart block observed in this patient is a recognized complication of surgical aortic valve replacement. The overall incidence of third-degree atrioventricular block after surgical aortic valve replacement is approximately 4.5%, with bicuspid aortic valve patients having a significantly higher incidence (6.5% versus 2.5%) compared to tricuspid aortic valve stenosis [15]. Although most cases require permanent pacemaker implantation, the conduction disturbance resolved spontaneously in our patient, consistent with the finding that new-onset third-degree atrioventricular block is not associated with worse long-term prognosis.

## Conclusions

This case describes the rare coexistence of critical bicuspid aortic stenosis, retroesophageal aberrant right subclavian artery causing dysphagia lusoria, and a contralateral subclavian artery aneurysm in a young adult. It highlights the importance of considering aortic arch anomalies in patients with congenital valvular disease who present with unexplained dysphagia. Computed tomography angiography with three-dimensional reconstruction was essential for accurate diagnosis and operative planning. In this patient, a single-stage hybrid approach combining carotid-subclavian translocation with surgical aortic valve replacement was successfully performed with resolution of dysphagia and a favorable early outcome. Management of the associated subclavian artery aneurysm was individualized because of the limited evidence and absence of definitive guideline recommendations, underscoring the importance of multidisciplinary decision-making and continued imaging surveillance.
